# Baseline Data of the Fungal Phytobiome of Three Sorghum (*Sorghum bicolor*) Cultivars in South Africa using Targeted Environmental Sequencing

**DOI:** 10.3390/jof7110978

**Published:** 2021-11-17

**Authors:** Gilmore T. Pambuka, Tonjock Rosemary Kinge, Soumya Ghosh, Errol D. Cason, Martin M. Nyaga, Marieka Gryzenhout

**Affiliations:** 1 Department of Genetics, University of the Free State, Bloemfontein 9310, South Africa; giles.pambuka@gmail.com (G.T.P.); GhoshS@ufs.ac.za (S.G.); 2 Department of Biological Sciences, University of Bamenda, Bambili P.O. Box 39, Cameroon; rosemary32us@yahoo.com; 3 Department of Animal Sciences, University of the Free State, Bloemfontein 9310, South Africa; CasonED@ufs.ac.za; 4 Next Generation Sequencing Unit and Division of Virology, University of the Free State, Bloemfontein 9310, South Africa; NyagaMM@ufs.ac.za

**Keywords:** sorghum, cultivars, plant tissues, substrates, above ground, below ground, illumina sequencing

## Abstract

Plant-associated fungi, or the mycobiome, inhabit plant surfaces above ground, reside in plant tissues as endophytes, or are rhizosphere in the narrow zone of soil surrounding plant roots. Studies have characterized mycobiomes of various plant species, but little is known about the sorghum mycobiome, especially in Africa, despite sorghum being one of the most important indigenous and commercial cereals in Africa. In this study, the mycobiome associated with above- and below-ground tissues of three commercial sorghum cultivars, as well as from rhizosphere and surrounding bulk soil samples, were sequenced using targeted sequencing with the Illumina MiSeq platform. Relative abundance differences between fungal communities were found between above-ground and below-ground niches, with most differences mostly in the dominant MOTUs, such as Davidiellaceae sp. (*Cladosporium*), Didymellaceae sp. 1 (*Phoma*), *Fusarium*, *Cryptococcus* and *Mucor*. Above-ground communities also appeared to be more diverse than below-ground communities, and plants harboured the most diversity. A considerable number of MOTUs were shared between the cultivars although, especially for NS5511, their abundances often differed. Several of the detected fungal groups include species that are plant pathogens of sorghum, such as *Fusarium*, and, at low levels, *Alternaria* and the Ustilaginomycetes. Findings from this study illustrate the usefulness of targeted sequencing of the ITS rDNA gene region (ITS2) to survey and monitor sorghum fungal communities and those from associated soils. This knowledge may provide tools for disease management and crop production and improvement.

## 1. Introduction

Sorghum (*Sorghum bicolor*) is the fifth most cultivated cereal crop in the world [1]. In Africa, sorghum is an important staple grain for millions of people, especially for rural communities [2]. Growing this crop has various advantages, including a comparable nutritional value to other cereal crops, such as maize, and better yields in dry and arid regions [2,3]. The latter is particularly true for South Africa, where sorghum is grown in drought-prone areas and considered a staple food in rural communities [4], thus providing better household food security than maize.

Plants interact with a dynamic community of microorganisms. These include fungi that colonize plant surfaces as epiphytes [5], endophytes inhabiting internal plant tissues [6] and pathogens [7]. Mycorrhiza are fungi that have specialized symbiotic relationships with plant roots [8]. In the rhizosphere (area of soil in direct contact and close proximity to roots), selected beneficial or detrimental fungi thrive [9]. Bulk soil are not close to roots and are inhabited by efficacious fungal communities that are classified based on their ecological functions [9]. While some fungi associated with plants may be pathogenic, others may form mutualistic, antagonistic or commensal associations with different plant hosts [10].

A plant is not subjected to one pathogen, one insect or one environmental factor, but rather to a complex ecosystem [11]. The phytobiome is a term encompassing the plant and its interactions with its surrounding ecosystem and communities of micro- and macro-organisms [12]. Identification of these microorganisms is the first step towards understanding the functions of the phytobiome, which could help yield more sustainable ways of increasing agricultural productivity and plant health [11].

Few studies have been performed using culture-independent techniques such as NGS to assess the natural occurrence and distribution of fungal communities in sorghum. These studies assessed fungal community diversity in the rhizosphere soils at different growth stages [13], mycorrhiza communities and genotype specificity in the roots, rhizosphere soils and bulk soils [14], communities in drought-stressed sorghum at different flowering stages in the leaf and root endosphere, rhizosphere soils and bulk soils [15], and seed mycobiomes [16]. Nothing has been conducted in relation to the entire plant community structure above- and below-ground. Culture-dependent methods mostly focused on potential pathogens such as *Fusarium* [4,17] and naturally occurring fungal communities [18]. Hidden plant-associated fungal communities are, thus, largely overlooked and poorly understood globally, despite their agronomical potential.

No culture-independent studies have been carried out in Africa, despite the importance of sorghum on the continent. In this study, targeted sequencing of the internal transcribed spacer (ITS) 2 region was used to characterize the fungal communities of three sorghum cultivars commonly planted in South Africa as first step for more targeted future studies. This presents a more rapid technique that would enable more elaborate surveys or monitoring in future compared to more conventional isolations. The aim of the study was thus to test the feasibility of the technique for this purpose and generate such data for sorghum for the first time in Africa. This was performed using different plant parts, both above and below soil level, as well as rhizosphere soils and bulk soils. The results will be important when creating baseline data for future studies comparing communities across various scenarios, such as planting regimes, or when studying community shifts or changes that can be exploited to increase agricultural production and the health of sorghum to benefit food security.

## 2. Materials and Methods

### 2.1. Sampling Sites and Collections

The sorghum plants, rhizosphere soil and soil used for environmental sequencing were sampled from the research field of the Grain Crops Institute, Agricultural Research Council in Potchefstroom, South Africa (26°43′43.16″ S—27°04′47.71″ E). Three commercial cultivars from Pannar Seeds, namely, PAN8076W, PAN8816 and NS5511, were planted in this study. The cultivars have outstanding yield performance, agronomic characteristics, and tannin content, and good tolerance to various pathogens. PAN8816 has red seeds, and low tannin and high malt content; PAN8706W has tan seeds, and low tannin and malt content; NS5511 has tan seeds, and high tannin and malt content.

There were six sorghum treatments in the trial (Table 1). The trial also included legume plant species (cowpea, dry bean, soybean, Bambara groundnut) and fallow soil, in addition to the sorghum cultivars (considered as a single sorghum treatment) (Table 1). This was done to provide variation in the agricultural ecosystem, allowing for the associated phytobiomes of a diversity of crops to be available for selection by sorghum plants, as well as fungi from soils and air in the same environment. Other trials that surrounded the trial of this study included maize and sunflower. The three repetitions were randomly distributed (Table 1). All three sorghum cultivars were planted in each sorghum treatment, with two rows per cultivar (total of six rows allocated for sorghum) randomized per treatment. Each treatment of sorghum was paired up with a treatment of legumes planted in the remaining rows. Standard agricultural practices and the maintenance of the trial were performed by the staff of the Agricultural Research Council.

The plants were sampled 7–8 weeks after sowing. Five healthy plants were randomly sampled for each cultivar for each of the three repeats per treatment, giving a total of 15 plants for each cultivar. Five randomly selected bulk soil samples per replicate plot were collected at a depth of 0–15cm and in the middle between two adjacent plants (in row spacing 25 mm). Rhizosphere soil sampled consisted of the soil adhering to the sorghum roots that were shaken off into separate plastic zip-lock bags. The bulk and rhizosphere samples also amounted to 15 per cultivar. The excavated plants, rhizosphere and bulk soil samples were transported in large cold plastic and zip-lock bags, respectively, to the laboratory for further processing at the University of the Free State, South Africa and to prevent cross-contamination.

### 2.2. Processing of Plant Material, Rhizosphere, and Soil Samples

Plant material from the sorghum cultivar samples were cut and separated for above-ground parts (seed, leaves, stems) and below-ground parts (roots). For each plant, twenty random seeds, leaves and roots, and the stem were washed thoroughly with tap water to remove soil and dust. After washing, all plant parts were surface-sterilized by sequential washing with 3% sodium hypochlorite for 3 min, sterile, distilled water for 1 min, 70% ethanol for 2 min, and sterile, distilled water for 1 min, followed by air-drying, chopping up into pieces (1 × 1 cm), and placement in 50 mL falcon tubes. The corresponding rhizosphere soil and bulk soil samples were homogenized and 20g were also transferred into 25 mL falcon tubes. All samples were freeze-dried, ground using a home mince grinder (thoroughly surface sterilized between samples) and mixed thoroughly. A representative amount (20 g) of the samples per treatment were transferred into 2 ml Eppendorf tubes and pulverized with 2-mm-diameter metal balls in a Qiagen Tissue Lyser II cell disrupter (Whitehead Scientific, Cape Town, South Africa) to randomly mix the sample contents. From these, at least 1 g of the plant tissue powder and soil per treatment were transferred to 2 ml Eppendorf tubes for DNA extraction.

### 2.3. Illumina DNA Sequencing

Total genomic DNA of the material from the different plant parts samples was extracted using the Nucleospin Plant Kit II (Macherey Nagel, Dueren, Germany) according to the manufacturer’s instructions. The Nucleospin Soil Kit (Macherey Nagel) was used to extract DNA from homogenized rhizosphere and bulk soil samples following the manufacturer’s instructions. The quantity and quality of extracted DNA was determined using a Nanodrop LITE spectrophotometer (Thermo Fisher Scientific, Waltham, MA, United States) and diluted to 10 ng/µL (1:10). The ITS (Internal Transcribed Spacer) 2 region of the fungal rDNA was amplified by PCR using the ITS3 (5′-GCATCGATGAAGAACGCAGC-3′) and ITS4 (5′-TCCTCCGCTTATTGATATGC-3′) primer set with overhanging Illumina adapters [19]. PCR amplification was carried out on each of the extracted samples in a final volume of 25 µL mixture containing 12.5 µL Kapa HiFi Ready-Mix DNA Polymerase (KAPA Biosystems, Lasec, Johannesburg, South Africa), 1.5 µL 10 mM ITS3 and ITS4 primers, 9 µl nuclease-free water and 2 µL template DNA. A negative control replaced DNA with PCR-grade water (Merck, Sigma Aldrich, Johannesburg, South Africa) to evaluate the presence of contaminants. PCR reactions were performed for each DNA sample using the G-Storm GS04822 thermal cycler (Somerton Biotechnology Centre, Somerton, UK) at 3 min for initial denaturation at 95 °C, followed by 25 cycles at 95 °C denaturation for 30 s, annealing at 58 °C for 30 s, extension at 72 °C for 30 s, and a final extension at 72 °C for 5 min. The PCR products were visualised under a UV light by 2% agarose gel electrophoresis with GelRed (Biotium, Inc, Fremnt, CA, USA) fluorescent nucleic acid dye. The amplicons from PCR reactions (20 seed, 20 leaves, 20 stem, 20 root, 15 rhizosphere and 15 bulk soil amplicons) were pooled at equal quantities for each cultivar per treatment according to their corresponding niche and substrate, and sent to the Next Generation Sequencing Unit, University of the Free State, South Africa, for Illumina sequencing preparations.

Pooled PCR amplicons were purified using the Agencourt AMPure XP bead clean up kit (Beckman Coulter, Atlanta, GA, USA) at the Next Generation Sequencing Unit. The final library was quantified using a Qubit 3.0 fluorometer (Life Technologies, Thermo Fisher Scientific). Validation was performed with 1µL of a 1:50 dilution of the final library on a Bioanalyzer DNA 1000 chip using the Bioanalyzer 2100 (Agilent Technologies, Santa Clara, CA, USA) to verify fragment size (200–300 bp). Purified amplicons were normalised and pooled together in equal concentrations (8 pmol) and paired-end sequencing (2 × 300 bp) was done using a MiSeq V3 (600 cycle) kit (Illumina Inc, San Diego, CA, USA) on an Illumina MiSeq platform (Illumina Inc, San Diego, CA, USA).

### 2.4. Cluster and Data Analysis

The sequence quality of forward and reverse sequences was assessed using FastQC v 0.11.8- Babraham Bioinformatics [20]. Prinseq lite version V0.20.4 was used for trimming and quality control of sequences to obtain an average quality score of ≥25 and a minimum sequence length of 200 bp [21]. Paired-end reads were merged using PEAR 0.9.6 [22] with default parameters. QIIME v1.9.1 was used to analyze paired-end reads [23]. Chimeric sequences were identified using USEARCH 6.1 [24] against the RDP ‘’Gold’’ database and were filtered out with QIIME using the identify_chimeric_seqs.py and filter_fasta.py commands, respectively. The default settings of two sequences per OTU as minimum were used to remove rare taxa. Sequences were clustered into Molecular Operational Taxonomic Units (MOTUs) against the ITS UNITE database (alpha version 12_11) released on 10.10.2017 [25] with the pick_open_reference_otus.py script, at a similarity threshold of 97% [26]. In some cases, where it is known that ITS sequence data cannot be confidently used to identify to species level, e.g., *Cladosporium*, *Phoma* and *Epicoccum* [16], MOTUs were only referred to on the family level. MOTU’s named by the pipeline with synonymous names, such as *Giberrella* currently known under *Fusarium*, were changed to the current name with the distinction indicated as sp. “x”, with x a numerical number based on the number of MOTUS for that genus.

For downstream analysis, QIIME version 1.9.1 was used to normalize the OTU-table using the CSS normalization option [27]. Fungal alpha diversity (i.e., abundance and richness) was calculated using Observed OTU indices and Shannon diversity metrics using the command alpha_rarefaction.py. Beta diversity was performed using Bray Curtis dissimilarity metrics and visualized with Principle Coordinates Analysis (PCoA) plots in RStudio [28] using the “plot_ordination” function in the “Phyloseq” package [29]. The software was used for additional analyses to visualize fungal diversity indices in different samples (rarefaction curves, and bar charts). The statistical significances of detected differences between cultivars and plant niches were compared using Permutational Multivariate Analysis of Variance (PERMANOVA) using the function “adonis2” in the vegan package. Molecular operational taxonomic units (MOTUs) containing more and less than 1% of the total sequences were separated using the ggplot function in the “Phyloseq” package for relative abundance (RA) graphs [29]. Unidentified MOTUs were not discarded and were included in the analysis. Names allocated by the pipeline were moderated in cases where they could be misleading, where it is known that the sequenced region cannot distinguish between species or genera, as is the case with the Didymellaceae and Mycosphaerellaceae, or in the case of older names, such as *Gibberella* in the case of *Fusarium* [16,30]. The tables incorporated for RA showed less diversity; therefore, Venn diagrams were plotted using the function in gplots in RStudio (https://CRAN.R-project.org/package=gplots, accessed on 25 June 2021) to show the total diversity between sorghum cultivars.

## 3. Results

### 3.1. Illumina DNA Sequencing

**Figure 1:**
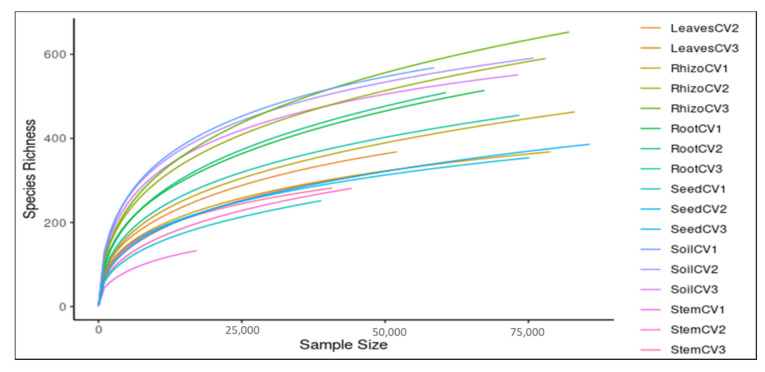
Rarefraction analysis of fungal community richness estimates based on sequences that passed the Phred quality score of 25. CV1, CV2, and CV3 correspond to sorghum cultivars PAN8076W, PAN8816 and NS5511, respectively.

After quality control, the average sequence length ranged from 251 to 300 bp at a base Phred quality score >25. A total of 4561 distinct MOTUs were assigned at a 97% sequence similarity. The number of sequences for each library ranged from 17128 to 83071, with individual MOTUs per sample ranging from 133 to 653 bp (Table 2). Rarefaction curves indicated that deeper sequencing is required to completely resolve fungal community diversity for some of the sampled niches (Figure 1).

### 3.2. Cluster and Data Analysis

#### 3.2.1. Molecular Operational Taxonomic Units Assignment

**Figure 2:**
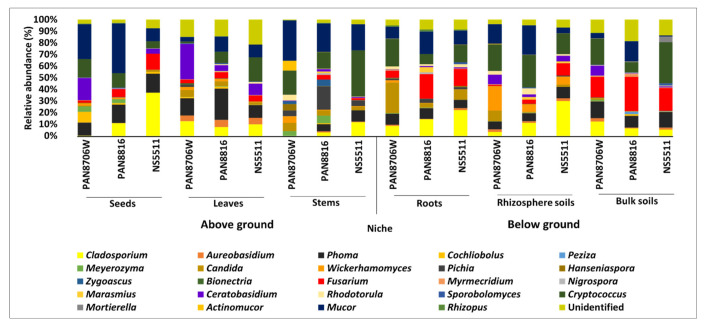
Relative abundance of fungal communities in the different sorghum cultivars at genus level (≥1%).

Analyses yielded diverse taxonomic classifications (Table 3 and Table 4, Supplementary Tables S1–S4; Figure 2, Supplementary Figure S1). Twenty-four of the assigned MOTUs were identified in the plant niches and between cultivars with RA ≥1% (Table 3; Figure 2). The results showed that most of the fungi in this group had a cosmopolitan distribution in cultivars and their respective substrates. Genera with low abundances had a total of 85 MOTUs assigned with abundance of less than 1% (0.1–1%) (Table 4). Their distribution was not as ubiquitous, with some fungal communities showing a specific or random occurrence.

In the Ascomycota, the Dothideomycetes represented one of the dominant phyla due to four MOTUs assigned with an RA greater than 1% (Table 3 and Figure 2), including Davidiellaceae sp. 1 (assigned in the pipeline as *Cladosporium*), *Aureobasidium*, Didymellaceae sp. 1 (assigned in the pipeline as *Phoma*), and *Cochliobolus*. The Sordariomycetes included five MOTUs, represented by the two most dominant MOTUs “*Nectriaceae*; other” and *Fusarium*, as well as *Myrmecridium*, and *Nigrospora*. BLAST searches of reads against GenBank (National Centre for Biotechnology Information) revealed that the MOTU with the assigned name of *Nectriaceae*_other, represented *Fusarium* (named here after as *Fusarium* sp. 1, while the other *Fusarium* MOTU was named *Fusarium* sp. 2). The basidiomycotan Tremellomycetes included the dominant MOTU *Cryptococcus*, and four others with relative abundances less than 1%. The assigned class Incertae_sedis in the Zygomycota was dominated by a single MOTU named *Mucor*.

A number of other classes in the Ascomycota and Basidiomycota could be found, with MOTUs having an RA higher and lower than 1% (Table 3 and Table 4). These included generic names such as *Alternaria*, *Cochliobolus*, *Bipolaris*, Didymellaceae sp. 2 (*Epicoccum*) and *Gibberella* (synonymous to *Fusarium*, here named *Fusarium* sp. 4). The least abundant phyla had MOTUs with less than 1% RA, namely two MOTUs in the Chytridiomycetes and three MOTUs in the Glomeromycetes.

#### 3.2.2. Relative Abundance among Different Substrates

The most dominant MOTUs, namely Davidiellaceae sp. 1 (*Cladosporium*), *Fusarium* spp. 1 and 2, Didymellaceae sp. 1 (*Phoma*), *Cryptococcus* and *Mucor,* mostly occurred across niches and plant parts (Table 3). Absences of these prominent MOTUs in some tissues, were random. In some cases, such as Davidiellaceae sp. 1 (*Cladosporium*), which was most dominant in the seed (26%), rhizosphere (21.7%) and roots (16.3%) of cultivar NS5511, RA values differed greatly between tissues, cultivars and niches. In other cases, such as Didymellaceae sp. 1 (*Phoma*), MOTUs had relatively similar RA levles. Based on RA, *Fusarium* sp. 1 had an apparent affinity more for above-ground tissues, whereas *Fusarium* sp. 1 were more abundant below ground.

MOTUS in the lower-abundance groups (≤1%) (Table 4) showed more sporadic RA patterns across niches, tissues and cultivars. The MOTUs Didymellaceae sp. 2 (assigned in the pipeline as *Epicoccum*) and *Alternaria* were observed across all samples, with *Alternaria* mostly in seeds and leaves, and rhizosphere and bulk soils. Possible trends could be detected for some, such as the MOTU *Fusarium* sp. 4 (assigned as *Gibberella*), which appeared only in plant-associated niches. Some MOTUs, such as *Alternaria*, *Cochliobolus* and *Bipolaris*, were mostly missing from stems and roots, but occurred in the majority of leave, stem and soil samples. A number of yeast species and mushroom forming taxa were also found. MOTUs in the Chytridiomycota and Glomeromycota were only present in rhizosphere soils and bulk soils.

**Figure 3:**
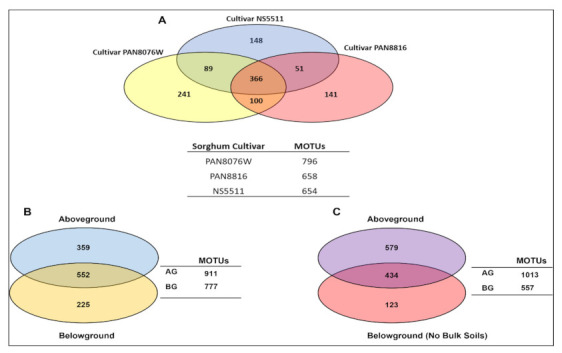
Venn diagram depicting shared and unique fungal Molecular Operational Taxonomic Units (MOTUs) in the three sorghum cultivars (**A**), combined cultivar MOTUs for all niches, above- and below-ground (**B**), and combined cultivar MOTUs above- and below-ground (**C**) (Bulk soils excluded).

Fungal community overlap and abundance differed between the above- and below-ground substrates for each of the sorghum cultivars (Table 3 and Table 4; Figure 3B,C). In the above-ground substrates, 911 MOTUs were found (Figure 3B), while 777 MOTUs were detected for below-ground communities (Figure 3B, Table 3 and Table 4). Above-soil-level tissues had more unique MOTUs than below-soil niches, which increased when bulk soils were excluded. Most MOTUs were plant-associated (excluding bulk soils), with more MOTUs still present above soil level. A high number of MOTUs were, however, still shared between above- and below-ground niches. The MOTUs detected in one of these niches could also be detected in another. For example, *Daldinia* was present in all the rhizosphere samples from each cultivar, but also detected in leaves and from no other substrate (Table 4).

When comparing the number of MOTUs between cultivars (Figure 3A), PAN8076W had 796 MOTUs compared to PAN8816 (658) and NS5511 (654). A considerable number of MOTUs were shared between the cultivars (366). PAN8076W and PAN8816 shared the highest number of MOTUs (100), followed by PAN8076W and NS5511 (89), and NS5511 and PAN8816 (51). The relative abundances of some of the dominant MOTUs between cultivars were similar, while with others there were large differences (Table 3). For instance, Didymellaceae sp. 1 (*Phoma*) had double the RA in the seed of NS5511 (11.3%) and PAN8816 (11%) than in PAN8076W (6.1%). In the Nectriaceae, *Fusarium* sp. 1 was most abundant in the stems of PAN8816 (55.8%) and NS5511 (38.3%) but absent in stems of PAN8076W, and also had higher RA in the seeds and stems of PAN8076W and PAN8816 than those of NS5511.

MOTUs known to be important for plant health were detected. The dominant MOTU groups *Fusarium* spp. 1 and 2, Didymellaceae sp. 1 (*Phoma*) and Davidiellaceae sp. include several species: plant pathogens and mycotoxin producers. Other pathogenic groups that were found included *Ceratobasidium*, *Alternaria*, *Bipolaris*, *Cochliobolus*, *Curvularia*, and Didymellaceae sp. 2 (*Epicoccum*). MOTUs assigned in the Ustilaginomycetes (*Pseudozyma*, *Sporisorium* and *Ustilago*) were mainly specific to seed at similar frequencies. Some species in the pathogenic genera can also be beneficial, such as in *Fusarium* (Falk et al., 1996; Horinouchi et al., 2007). Others that are beneficial with RA ≤1% included the MOTU *Glomus*.

#### 3.2.3. Grouping of Fungal Communities in Plant Tissues and Cultivars

**Figure 4:**
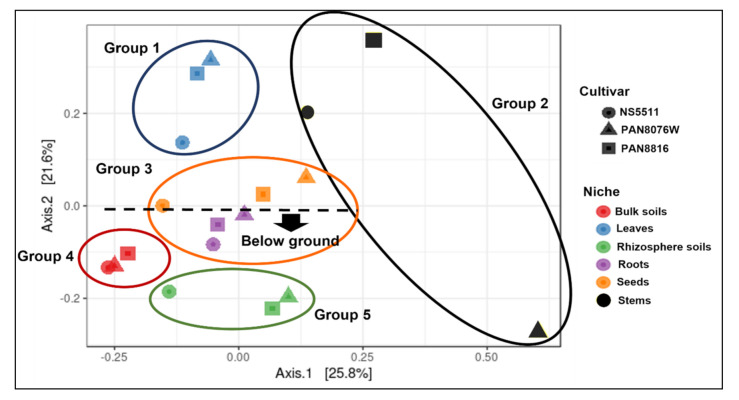
Principal coordinate analysis of fungal communities of the different sorghum cultivars and niches.

Principal coordinate analysis indicating possible distinct groupings between the various tissues of the plants from the above- and below-ground niches of the three sorghum cultivars, and their soil types, showed principal component 1 and principal component 2 at 25.8% and 21.6% of the variation, respectively. The resulting analyses separated the cultivars and niches into five groups (Figure 4) that represented the different niches, except for group 3, which included seeds and roots. Distinct groupings between plant niches were also shown to be statistically significant (*p =* 0.001), while no significant difference (*p* = 0.321) was observed between cultivars (Table 5). However, Venn diagrams (Figure 3) showed that cultivars shared MOTUs, with cultivar NS5511 (unique in having a high tannin content) being grouped more distantly from the other two cultivars in most substrates in the PCOA analyses, except in the bulk soil and roots. The separation was especially evident in the leaves (Group 1) and rhizospheres (Group 5).

Overall, the above- and below-ground niches for cultivar PAN8076W, PAN8816 and NS5511 were clustered separately. For above-ground, the groupings of the plant tissue were more separated from each other compared to groupings of below-ground plant tissue, rhizospheres, and soils, which were relatively closer together. Furthermore, significant differences (*p =* 0.001) were observed between the above- and below-ground niches (Table 5). The leaves (Group 1) of PAN8076W and PAN8816 grouped more closely together than the leaves of NS5511. The seed (Group 3) also followed a similar trend, with those of NS5511 slightly separate from the cultivars in the same niche. Stems (Group 2) grouped completely distinctly from each other, especially those of PAN8076W that were completely separated from the other cultivars and separate from the other above-ground niches. For below-ground niches, bulk soils, rhizosphere soils and roots were each separate, but grouped relatively close together. The bulk soils of cultivars NS5511, PAN8076W, and PAN8816 (Group 4) grouped closely together, whereas with the rhizosphere soils (Group 5) cultivars PAN8076W and PAN8816 grouped more closely together, while the rhizosphere soil (NS5511) was grouped more closely to the bulk soils. The roots of PAN8076W, PAN8816 and NS5511 grouped together, but, interestingly, they were more closely clustered to the above-ground seed niche of the same cultivars in the same group (Group 3).

## 4. Discussion

In this study, targeted sequencing was used to successfully characterize the fungal mycobiome of three commercial sorghum cultivars (PAN8076W, PAN8816, and NS5511) grown under field conditions in South Africa. This first approach in Africa to profile the entire fungal mycobiome for the above- and below-ground niches of sorghum cultivars provided a useful baseline of the sorghum mycobiome, albeit only from a single location, which can be used in future studies on more specific aspects. The results showed that the mycobiomes across the niches were dominated by Davidiellaceae sp. 1 (*Cladosporium*), Didymellaceae sp. 1 (*Phoma*), *Fusarium*, *Cryptococcus* and *Mucor*. These MOTUs generally occurred across tissue types, niches and cultivars. Plant pathogens such as *Ceratobasidium*, the sexual form of *Rhizoctonia* species causing seed rot, root rot, crown rot, and stem rot of various crops [31] were detected, even if some were present in low relative abundances. Above-ground plant tissues harboured more MOTUs than below-ground tissues and soils combined. A possible selection of fungal communities was suggested between cultivars, with those of NS5511 appearing more distinct, but communities were not significantly different between cultivars.

The occurrence and distribution of fungal genera such as *Fusarium* and *Phoma* have been reported to occur naturally as endophytes in sorghum grown in Burkina Faso [32]. In another study, *Fusarium* was commonly isolated as an endophyte in ten major growing areas of sorghum in India [33]. Using next-generation sequencing, these genera were observed in different plant tissues, bulk soils and rhizosphere soils for sorghum [13,15,16]. *Cladosporium*, *Phoma*, *Fusarium* and *Cryptococcus* were also observed in the seed of different sorghum cultivars grown in South Africa [16], including PAN8976W, PAN8816 and NS5511, used in this study for planting. However, these genera and families are also ubiquitous and known cosmopolitans, found in various environments, substrates and hosts [34].

Possible trends or patterns observed in this study must be confirmed in future research, where more appropriate sampling and sequencing replicates were performed to enable statistical verification. The extent to which detection could be due to chance infections that do not necessarily represent colonization and selection has not been established in this study [35,36]. More extensive sampling and sequencing is needed to better delimit the above- and below-ground communities. The factors that could possibly drive differences between above- and below-ground microbial communities, as has been shown in other studies, such as environmental factors, dissemination strategies and their different functional roles, are still unclear [6,37,38]. The selection of fungal communities at the root-rhizosphere-soil interface due to the release of exudates [39,40] have been observed for sorghum cultivars [13], but this needs to be studied in more detail in the South African context. The extent of specialization and diversification in different plant tissues that has been reported from other plants [41,42] has been hinted at based on the results of the study. Plant genotype is an important factor in structuring the diversity and selection of microbial communities [10,43], and has been shown in sorghum [13,15] and possibly indicated in this study. Understanding these various interactions could prove useful, providing knowledge on maintaining and stabilizing various ecological processes [44,45].

Vertical transmission of fungal endophytes has been shown in various plant species such as forbs [46], perennial ryegrass [47], and is especially known in grasses involving specialised fungi in the Clavicipitacecae [35]. Proving vertical transmission is difficult, since it must be established that these fungi did not already exist in the experimental plants [46,48]. The PCOA plot showed a close grouping of the fungal communities of seed and root tissues, especially for the cultivars PAN8816 and NS5511. This could possibly indicate vertical transmission between the seeds and roots, where fungi in the seeds also occurred in the roots, suggesting they were transferred from seeds. However, further scrutiny of the MOTUs showed that the shared MOTUs represented cosmopolitan fungal groups, while less abundant, possibly more specific, MOTUs were not present in either roots or seeds.

*Fusarium* was represented by four MOTUs in this study, of which two were among the most dominant detected MOTUS. *Fusarium* species occur in various habitats with diverse roles such as endophytes, saprobes in soil, pathogens of plants, humans and animals or beneficials to plants [49,50]. In sorghum, *Fusarium* species are known as phytopathogens and producers of mycotoxins [4,17,51]. *Fusarium* species that cause root rot, stalk rot, ear rots and those which cause grain mold are of major concern, as they significantly reduce yield in fields and contaminate stored grain through the production of mycotoxins, which could pose health risks to humans and animals [52,53]. The diversity and identity of *Fusarium* species infecting sorghum is still limited, especially in Africa [52].

MOTUs, assigned as *Cladosporium* (Davidielleace) and Didymellaceae sp. 1 (*Phoma*), were among the most dominant MOTUs. Species in both these families are geographically prevalent and found in diverse ecological niches, most importantly as plant pathogens on important food crops [54,55]. Other high-throughput sequencing studies also found *Cladosporium* and *Phoma* to be prominent or present in various plant tissue, such as seed, leaves, and roots, and in rhizosphere soils and bulk soils of sorghum [15,16]. Numerous genera exist in the Didymellaceae that are well known to affect sorghum. An example is *Epicoccum sorghinum* (previously named *Phoma sorghina*), a contaminant of sorghum grain, especially at the pre-harvest stage in the field, reducing yield. In addition, *E. sorghinum* is known to produce mycotoxins that can be harmful to humans and animals [56]. Likewise, numerous genera in *Cladosporium* have been reported to be important plant pathogens, such as *C. cladosporioides,* associated with the grain mould complex in sorghum and typical leaf spot discolorations in the field [57].

In sorghum, *A. alternata* are commonly associated with the grain mold complex, affecting the quality of sorghum grain both pre- and post-harvest, while the same species has been known to cause leaf spot [58,59], including in South Africa [60]. *Alternaria* had relative abundances below 1% in the seed harvested from the field from three of the six used cultivars, while also having low RA values in the other plant tissues and substrates. Curiously, *Alternaria* was found to be dominant (above 1%) in the commercial seeds of six sorghum cultivars used to establish the current trial [16].

An important group of plant pathogenic fungi, although occurring in low abundances, was the Ustilaginomycetes. Three MOTUs assigned as *Thecaphora*, *Sporisorium* and *Ustilago*, were detected, and they occurred in the seed. Members in this group are known smut fungi pathogenic to cereals and other crop plants [61,62]. In sorghum, smut fungi known to colonize the kernels of the crop include species, such as *Sporisorium sorghi* (kernel smut), *Sporisorium ehrenbergii* (long smut), and *Sporisorium cruentum* (loose kernel smut), which cause severe losses in yield [63,64].

*Cryptococcus* was omnipresent and dominant in all samples. *Cryptococcus* species are yeast saprobes from niches such as the phylloplane, stems, leaves [65], and often a dominant fungal group in soils [66]. The genus is among the most prominent fungal molecular operational taxonomic units in culture independent studies [67,68]. Generally, the genus is not considered phytopathogenic, but as a human pathogen [69]. Some species have also been proven as biocontrol agents of plant pathogenic fungi and post-harvest diseases in various plant species, such as wheat (*Triticum aestivum*), and mandarin orange (*Citrus reticulata*) [70,71].

*Mucor* was the most abundant Zygomycete identified in the plant niche of the sorghum cultivars. Species in *Mucor* are ubiquitous in nature, especially soils, and predominantly found as saprotrophs or endophytes [72]. They are predominantly associated with humans as pathogens [73], but have also been reported to be harmful in plants such as *Mucor piriformis,* causing rot in field and post-harvest on cherries [74]. Other MOTUs that were found in lower abundances, such as *Rhizopus*, are known to cause rots in other crop, such as grapes, while *Rhizopus stolonifer* has been linked to the seed of sorghum [75]. Some species are known to have biocontrol properties, such as enhancing growth in plants such as *Arabidopsis arenosa* [76].

Only a single MOTU representing arbuscular mycorrhizal fungi (AMF) [77], namely, *Glomus*, was detected. The MOTU was absent in roots and detected in soils in low abundance. Previous studies have found AMF communities in the roots, rhizosphere and soil of sorghum [14,15]. The Aasence of AMF fungi in this study could be due to agricultural practices or bias introduced by the primers used, because other studies found the primer sets 5.8SFun and ITS4Fun [14,15,78] to match well with all Glomeromycotina lineages.

## 5. Conclusions

Sorghum is an important food crop to rural communities in Africa, but there are still substantial knowledge gaps regarding the fungal communities associated with the crop. Such knowledge is invaluable because fungal communities play a fundamental role in ecosystem services, recycling nutrients as well as serving as decomposers, plant pathogens and saprophytes, and providing beneficial communities to the plant as endophytes, epiphytes, mycorrhiza, and general soil-inhabiting communities [9,79]. The high fungal diversity between the niches and cultivars in this study indicates that these fungal communities could have potential applications in improving the performance of sorghum in different environments. For example, whether these communities can be exploited to control pathogenic species could be investigated. However, further investigation is needed into the function and complex interactions in the sorghum holobiont. Such data can easily be generated by targeted environmental sequencing and used to improve the productivity and performance of the crop in agricultural setups, especially for the rural community.

## Figures and Tables

**Table 1:** Schematic representation of the field layout. PAN8706W, PAN8816, and N5511 are the sorghum cultivars used in this study. ‘× 2′ and ‘× 6′ depicts 2 and 6 rows of cultivated sorghum and leguminous plants, respectively. The similar color code indicates similar plant combinations with alternating orders.

PAN8076W × 2	PAN8076W × 2	PAN8076W × 2	PAN8076W × 2	PAN8076W × 2	PAN8076W × 2
PAN8816 × 2	PAN8816 × 2	PAN8816 × 2	PAN8816 × 2	PAN8816 × 2	PAN8816 × 2
NS5511 × 2	NS5511 × 2	NS5511 × 2	NS5511 × 2	NS5511 × 2	NS5511 × 2
**Dry bean × 6**	**Cowpea × 6**	**Soybean × 6**	**Sorghum (8816) × 6**	**Fallow × 6**	**Bambara groundnut × 6**
NS5511 × 2	NS5511 × 2	NS5511 × 2	NS5511 × 2	NS5511 × 2	NS5511 × 2
PAN8076W × 2	PAN8076W × 2	PAN8076W × 2	PAN8076W × 2	PAN8076W × 2	PAN8076W × 2
PAN8816 × 2	PAN8816 × 2	PAN8816 × 2	PAN8816 × 2	PAN8816 × 2	PAN8816 × 2
**Fallow × 6**	**Sorghum (8816) × 6**	**Dry bean × 6**	**Bambara groundnut × 6**	**Soybean × 6**	**Cowpea × 6**
PAN8816 × 2	PAN8816 × 2	PAN8816 × 2	PAN8816 × 2	PAN8816 × 2	PAN8816 × 2
NS5511 × 2	NS5511 × 2	NS5511 × 2	NS5511 × 2	NS5511 × 2	NS5511 × 2
PAN8076W × 2	PAN8076W × 2	PAN8076W × 2	PAN8076W × 2	PAN8076W × 2	PAN8076W × 2
**Soybean × 6**	**Bambara groundnut × 6**	**Fallow × 6**	**Dry bean × 6**	**Cowpea × 6**	**Sorghum (8816) × 6**

**Table 2:** Sequencing results and number of Molecular Operational Taxonomic Units (MOTUs) for the three sorghum cultivars investigated, before and after quality control (QC).

Cultivar	Sample	Number of Reads before QC (bp)	Number of Reads after QC (bp)	Total MOTUs
PAN8076W	Seeds	56,429	38,879	252
	Leaves	77,662	59,724	341
	Stems	78,912	17,128	133
	Roots	80,959	67,341	514
	Rhizosphere soils	93,649	83,071	463
	Bulk soils	77,500	58,539	568
PAN8816	Seeds	86,996	75,159	354
	Leaves	72,655	52,055	368
	Stems	65,161	44,167	281
	Roots	79,755	60,665	509
	Rhizosphere soils	95,588	78,004	590
	Bulk soils	91,469	75,901	591
NS5511	Seeds	103,073	85,644	386
	Leaves	85,671	78,788	368
	Stems	79,208	40,787	282
	Roots	82,309	73,416	455
	Rhizosphere soils	93,861	82,108	653
	Bulk soils	88,034	73,200	551

**Table 3:** Summary of the relative abundance (%) of Molecular Operational Taxonomic Units (MOTUs) taxa that were assigned at genus level with abundances ≥1% for the different sorghum cultivars and above- and below-ground niches.

Molecular Operational Taxonomic Units	Above Ground									Below Ground								
	Seeds			Leaves			Stems			Roots			Rhizosphere Soils			Bulk Soils		
	PAN8076W	PAN8816	NS5511	PAN8076W	PAN8816	NS5511	PAN8076W	PAN8816	NS5511	PAN8076W	PAN8816	NS5511	PAN8076W	PAN8816	NS5511	PAN8076W	PAN8816	NS5511
**Ascomycota**																		
**Dothideomycetes**																		
Davidielleace sp. 1 (*Cladosporium*)		**7.7**	26.0 *	**6.4**	2.7	4.7		1.2	6.3	6.1	9.9	16.3 *	2.7	8.0	*21.7* *	6.7	3.2	3.2
*Aureobasidium* sp. 1				2.5	2.2	2.6						1.1	1.6		1.7	1.5		
Didymellaceae sp. 1 (*Phoma*)	**6.1**	**11.0**	**11.3**	**7.5**	**9.5**	**5.2**		**2.6**	**5.2**	**6.7**	**6.6**	**5.2**	**5.4**	**5.1**	**7.0**	**7.7**	**5.1**	**8.1**
*Cochliobolus*	**5.1**		1.2															
Saccharomycetes																		
*Meyerozyma* sp. 1	2.5	2.4					4.0	2.7										
*Candida* sp. 1				3.0	1.5	1.0	7.0	1.9	1.6	18.9 *	2.5	6.5	7.2		1.4			
*Wickerhamomyces* sp. 1	1.1			1.3			**5.5**			**1.5**			**16.8**	**4.7**	**4.9**			
*Pichia* sp. 1							4.8	8.4	2.6		2.3	1.4						
*Hanseniaspora* sp. 1							5.4											
*Zygoascus* sp. 1							2.5											
**Sordariomycetes**																		
*Fusarium* sp. 1 (Nectriaceae other)	**10.1**	**13.8**	**6.1**	31.5 *	28.6 *	15.0 *		55.8^*^	38.3 *	**14.7**	**13.5**	**10.5**	**3.5**	**3.6**	**3.2**	**6.9**	**9.7**	**7.7**
*Fusarium* sp. 2	1.1	4.9	9.6	1.6	1.9	2.4		1.7		4.5	14.7 *	10.7		2.5	7.5	9.5	14.7 *	11.7
*Myrmecridium*																1.3		
*Nigrospora*											1.1							
**Basidiomycota**																		
**Agaricomycetes**																		
*Marasmius*											3.2			1.5				
*Ceratobasidium*	**10.4**		**3.0**	**15.3**	1.8	4.6							6.7	1.7	3.5	4.6		
**Microbotryomycetes**																		
*Rhodotorula*							4.9			1.7	1.1	1.9	2.5	3.6				
*Sporobolomyces*												1.1						
Tremellomycetes																		
*Cryptococcus*	**8.7**	**8.8**	**4.3**		**3.5**	**9.9**	**20.1**	**6.0**	**21.3**	**17.0**	**5.9**	**11.0**	18.0 *	20.0 *	**12.9**	11.9 *	**0.4**	21.2 *
**Zygomycota**																		
**Incertae_sedis**																		
*Mortierella*																		2.9
*Actinomucor*							8.4											
*Mucor*	16.3 *	30.0 *	**7.8**	**2.0**	**4.6**	**5.1**	33.7 *	**10.3**	**12.0**	**7.2**	**13.5**	**8.9**	**13.3**	**18.0**	**3.5**	**2.3**	**8.7**	
*Rhizopus*											1.2							
**Unidentified**																		
Unidentified	**1.9**	**2.0**	**5.1**	**7.4**	**5.0**	**10.1**		**1.2**	**2.1**	**3.5**	**5.9**	**6.1**	**3.0**	**3.4**	**4.9**	**6.0**	**9.2**	**8.1**

Numerical values in bold are relative abundances (RA) 5% and above, whilst numbers in bold and italics are above 20%. Numerical values followed by an asterisk indicate MOTUs, which were the highest in the niche, whilst MOTUs, which were RA 1% and below, are left blank in the table. Names are used as assigned by the UNITE database.

**Table 4:** Summary of the relative abundance (%) of Molecular Operational Taxonomic Units (MOTUs) that were assigned names with abundances ≤1% for the different sorghum cultivars and above- and below-ground niches.

Molecular Operational	Above Ground									Below Ground								
Taxonomic Units	Seeds			Leaves			Stems			Roots			Rhizosphere Soils			Bulk Soils		
	PAN8097W	PAN8816	NS5511	PAN8097W	PAN8816	NS5511	PAN8097W	PAN8816	NS5511	PAN8097W	PAN8816	NS5511	PAN8097W	PAN8816	NS5511	PAN8097W	PAN8816	NS5511
**Ascomycota**																		
**Dothideomycetes**																		
*Neofusicoccum*																		0.1
Mycosphaerellaceae sp.		0.2	**0.5**	0.1	0.1	0.2			0.1	0.2	0.1	0.1	0.1	0.1	**0.3**	0.1		
*Cladosporium* sp. 2	0.1																	
*Aureobasidium* sp. 2		0.1						**0.4**	**0.3**	**0.8**	0.2			**0.9**			**0.3**	**1**
*Rhizopycnis*																0.1		
Incertae sedis sp.														0.1	0.1	0.2	0.1	0.1
*Stagonospora*														0.1				
Pleosporaceae sp.	0.1		**0.3**															
*Alternaria*	0.1	0.1	0.2	0.1	**0.3**	0.2				0.2			0.1	0.1	0.2	0.2	**0.1**	**0.1**
*Bipolaris*			**0.5**		0.1	0.1							0.1		0.1	0.1	0.1	**0.3**
*Cochliobolus*		**0.9**		**0.5**	**0.6**	**0.1**		**0.3**			0.1					0.1	**0.8**	0.2
*Curvularia*																	0.1	0.1
Didymellaceae sp. 2 (*Epicoccum*)		0.1	0.1	**0.4**	0.1	0.1			0.1	0.2	0.1	0.1	0.2	0.2	0.2	0.2	0.1	0.1
Unidentified																		0.1
**Eurotiomycetes**																		
*Exophiala*									0.2	0.2	0.2	0.1			0.2	0.1	**0.4**	**0.4**
*Rhinocladiella*							**0.4**											
Orbiliomycetes																		
*Arthrobotrys*																	0.2	0.2
*Dactylella*																0.1		
**Pezizomycetes**																		
*Peziza*																0.1	**1**	**0.3**
Unidentified sp.															0.1			
**Saccharomycetes**																		
*Meyerozyma* sp. 2			0.1	0.1	0.1	0.1			0.1	**0.3**	**0.3**	0.1	0.2	**0.3**		**0.6**		
*Candida* sp. 2	**0.4**	0.1	**1**			**1**								**0.4**		**0.8**		
*Cyberlindnera*							0.1	0.1	0.1	***0.2***				**0.3**				
*Wickerhamomyces* sp. 2		**0.9**	**0.2**		**0.7**	**0.2**		**0.3**	**0.3**		0.2	0.1					0.2	
*Clavispora*						0.1									0.2			
*Pichia* sp. 2		0.1	0.1	**1**	0.1					**0.8**			0.1	0.2	**0.7**	0.1		
*Hanseniaspora* sp. 2	**0.3**					0.1			0.1	**5**		**0.7**						
*Zygoascus* sp. 2									0.1	0.1								
**Sordariomycetes**																		
Diaporthales sp.														**0.2**		0.1	0.1	0.1
*Bionectria*				**0.6**						0.1	0.1							
*Fusarium* sp. 3									**0.9**				**0.8**					
*Fusarium* sp. 4 (assigned as *Gibberella*)	0.1	0.2	**0.5**	**0.3**	0.1	0.2		0.2	**0.4**	0.2	0.1	0.1		**0.3**	0.1			
*Myrmecridium*				0.1	0.1					0.1						**0.5**		0.1
*Sphaeronaemella*			0.1		0.1	0.1												
Microascaceae sp.																	0.1	
*Colletotrichum*																**0.2**		
Sordariales sp.																**0.8**	**0.6**	
Lasiosphaeriaceae sp.												0.1					**0.4**	
Unidentified												0.1			0.1	**0.3**	**0.9**	
*Nigrospora*		0.1			0.2	0.2		**0.6**		0.2	**1**				0.2		0.1	
*Microdochium*				0.1												0.1	**0.3**	0.1
*Daldinia*				0.1	0.1								0.1	0.2	0.1			
**Basidiomycota**																		
**Agaricomycetes**																		
*Agaricus*												**0.7**			0.1			
*Agrocybe*										0.1								
*Conocybe*																	0.1	
*Panaeolus*															0.1			
*Marasmius*					0.1			**0.6**		**0.3**		0.2	0.2		**1**			0.1
*Coprinellus*										0.1						0.1		
*Coprinopsis*																	0.1	
*Athelia*													0.1	0.1	0.1			
*Ceratobasidium*		0.2					**0.5**	**0.9**	**0.5**	0.2	**0.7**	**0.8**					0.1	**0.6**
*Thanatephorus*														0.2		0.2		
Corticiaceae sp.										0.1							0.1	
*Waitea*		0.1																
*Gloeophyllum*																0.1	0.1	0.1
Sebacinaceae sp.												0.1		0.1	0.1	0.1		
**Cystobasidiomycetes**																		
*Occultifur*							0.1							0,1				
**Exobasidiomycetes**																		
Unidentified sp.																		
**Microbotryomycetes**																		
Sporidiobolales sp.						0.1		**0.3**	0.1	0.1	0.2	**0.3**		0.1	0.1	**0.3**	**0.3**	**0.9**
*Rhodotorula*	0.1	0.1	0.1		**0.3**	**0.7**		0.1	0.2						**0.9**	**0.3**	**0.3**	**0.1**
*Sporobolomyces*		**0.4**		***0.2***	**0.3**	0.1	0.1				0.1		**0.2**	**0.6**	0.1	0.2	0.2	
Unidentified sp.		0.1						0.1	0.2							0.1		0.1
**Tremellomycetes**																		
*Cystofilobasidium*										**0.3**			**0.6**					
Tremellaceae														0.1				
*Dioszegia*																	0.1	**0.2**
*Tremella*				0.1														
**Ustilaginomycetes**																		
*Thecaphora*																	0.1	
Ustilaginaceae			**0.3**															
*Pseudozyma*		0.2	0.2															
*Sporisorium*	0.1	0.1	0.2					0.1										
*Ustilago*	0.2	**0.3**	0.2													0.1		
Unidentified			**0.9**			**0.3**					**0.6**			0.1	**0.7**		0.1	0.1
**Chytridiomycota**																		
Rhizophydiaceae sp.																	**0.5**	
*Spizellomyces*																	0.1	0.1
**Glomeromycota**																		
*Glomus*																	0.1	
Glomeraceae sp.																	0.1	
Unidentified sp.														0.1	0.1		0.1	0.1
**Zygomycota**																		
*Mortierella*												0.1		0.2		0.2	**0.3**	
Choanephoraceae sp.											0.2						0.2	
Mucoraceae sp.	0.2	0.1			0.1		**0.3**	0.1		0.2	**0.6**	0.2	0.1	**0.6**	**0.7**			
*Actinomucor*										0.2			**0.4**					
*Rhizomucor*					0.1													
*Rhizopus*	**0.3**	**0.3**		0.1	0.2		**0.6**	0.2		**0.8**		**0.8**	0.1	0.1		0.2	0.1	0.1

Numerical values in bold are relative abundances (RA) between 0.3% 1% and above, whilst MOTUs which were RA > 1% and with zero values were left blank in the table. Names are used as assigned by the UNITE database.

**Table 5:** Comparison between cultivars, plant niche, and above- vs. below-ground niches according to the Adonis permutation test.

Compared Categories	D.F	Sum of Sqs	R2	*p*-Value
Cultivar	2	0.38101	0.13015	0.321
Plant niche	5	1.7338	0.59222	0.001
Above-ground vs. below-ground niches	1	0.56049	0.19145	0.001

## Data Availability

The sequencing data generated in this study are deposited in NCBI with accession numbers.

## Supplementary Materials

The following are available online at https://www.mdpi.com/article/10.3390/jof7110978/s1, Figure S1: Bar charts of mycobiomes detected in the three sorghum cultivars (PAN8706W, PAN8816, N5511) at different taxonomic ranks: (A) Phyla, (B) Classes, (C) Orders, (D) Families. Table S1: Summary of the relative abundance (%) of Molecular Operational Taxonomic Units (MOTUs) taxa that were assigned at Phyla level for the different sorghum cultivars (above- and below-ground niche). Table S2: Summary of the relative abundance (%) of Molecular Operational Taxonomic Units (MOTUs) taxa that were assigned at Class level for the different sorghum cultivars (above- and below-ground niche). Table S3: Summary of the relative abundance (%) of Molecular Operational Taxonomic Units (MOTUs) taxa that were assigned at Order level for the different sorghum cultivars (above- and below-ground niche). Table S4: Summary of the relative abundance (%) of Molecular Operational Taxonomic Units (MOTUs) taxa that were assigned at Family level for the different sorghum cultivars (above and below ground niche).

## References

[1] Mundia C.W., Secchi S., Akamani K., Wang G. (2019). A Regional Comparison of Factors Affecting Global Sorghum Production: The Case of North America, Asia and Africa’s Sahel. Sustainability.

[2] Hadebe S.T., Modi A.T., Mabhaudhi T. (2017). Drought Tolerance and Water Use of Cereal Crops: A Focus on Sorghum as a Food Security Crop in Sub-Saharan Africa. J. Agron. Crop Sci..

[3] Motlhaodi T., Bryngelsson T., Chite S., Fatih M., Ortiz R., Geleta M. (2018). Nutritional variation in sorghum [Sorghum bicolor (L.) Moench] accessions from southern Africa revealed by protein and mineral composition. J. Cereal Sci..

[4] Beukes I., Rose L.J., Shephard G.S., Flett B.C., Viljoen A. (2017). Mycotoxigenic Fusarium species associated with grain crops in South Africa-A review. S. Afr. J. Sci..

[5] Bacon C.W., White J.F. (2016). Functions, mechanisms and regulation of endophytic and epiphytic microbial communities of plants. Symbiosis.

[6] Hardoim P.R., van Overbeek L.S., Berg G., Pirttilä A.M., Compant S., Campisano A., Döring M., Sessitsch A. (2015). The Hidden World within Plants: Ecological and Evolutionary Considerations for Defining Functioning of Microbial Endophytes. Microbiol. Mol. Biol. Rev..

[7] Agrios G. (2004). Plant Pathology.

[8] Johnson N.C., Jansa J. (2017). Mycorrhizas: At the Interface of Biological, Soil, and Earth Sciences. Mycorrhizal Mediation of Soil: Fertility, Structure, and Carbon Storage.

[9] Frac M., Hannula S.E., Belka M., Jȩdryczka M. (2018). Fungal biodiversity and their role in soil health. Front. Microbiol..

[10] Schlaeppi K., Bulgarelli D. (2015). The Plant Microbiome at Work. Mol. Plant-Microbe Interact..

[11] Beans C. (2017). Core Concept: Probing the phytobiome to advance agriculture. Proc. Natl. Acad. Sci. USA.

[12] Leach J.E., Triplett L.R., Argueso C.T., Trivedi P. (2017). Leading Edge Review Communication in the Phytobiome. Cell.

[13] Schlemper T.R., van Veen J.A., Kuramae E.E. (2018). Co-Variation of Bacterial and Fungal Communities in Different Sorghum Cultivars and Growth Stages is Soil Dependent. Microb. Ecol..

[14] Gao C., Montoya L., Xu L., Madera M., Hollingsworth J., Purdom E., Hutmacher R.B., Dahlberg J.A., Coleman-Derr D., Lemaux P.G. (2019). Strong succession in arbuscular mycorrhizal fungal communities. ISME J..

[15] Gao C., Montoya L., Xu L., Madera M., Hollingsworth J., Purdom E., Singan V., Vogel J., Hutmacher R.B., Dahlberg J.A. (2020). Fungal community assembly in drought-stressed sorghum shows stochasticity, selection, and universal ecological dynamics. Nat. Commun..

[16] Kinge T., Cason E., Valverde A., Nyaga M., Gryzenhout M. (2019). Endophytic seed mycobiome of six sorghum (Sorghum bicolor) cultivars from commercial seedlots using an Illumina sequencing approach. Mycsophere.

[17] Chala A., Degefu T., Brurberg M.B. (2019). Phylogenetically diverse Fusarium species associated with sorghum (Sorghum bicolor L. Moench) and finger millet (Eleusine coracana L. Garten) grains from Ethiopia. Diversity.

[18] Bamisile B.S., Dash C.K., Akutse K.S., Keppanan R., Wang L. (2018). Fungal Endophytes: Beyond Herbivore Management. Front. Microbiol..

[19] Caporaso J.G., Lauber C.L., Walters W.A., Berg-Lyons D., Huntley J., Fierer N., Owens S.M., Betley J., Fraser L., Bauer M. (2012). Ultra-high-throughput microbial community analysis on the Illumina HiSeq and MiSeq platforms. ISME J..

[20] Andrews S. FastQC A Quality Control Tool for High Throughput Sequence Data. http://www.bioinformatics.babraham.ac.uk/projects/fastqc/.

[21] Schmieder R., Edwards R. (2011). Quality control and preprocessing of metagenomic datasets. Bioinformatics.

[22] Zhang J., Kobert K., Flouri T., Stamatakis A. (2014). PEAR: A fast and accurate Illumina Paired-End reAd mergeR. Bioinformatics.

[23] Caporaso J.G., Kuczynski J., Stombaugh J., Bittinger K., Bushman F.D., Costello E.K., Fierer N., Peña A.G., Goodrich J.K., Gordon J.I. (2010). QIIME allows analysis of high-throughput community sequencing data. Nat. Methods.

[24] Edgar R.C. (2010). Search and clustering orders of magnitude faster than BLAST. Bioinformatics.

[25] Nilsson R.H., Larsson K.-H., Taylor A.F.S., Bengtsson-Palme J., Jeppesen T.S., Schigel D., Kennedy P., Picard K., Gï Ockner F.O., Tedersoo L. (2018). The UNITE database for molecular identification of fungi: Handling dark taxa and parallel taxonomic classifications. Nucleic Acids Res..

[26] Solanki M.K., Abdelfattah A., Britzi M., Zakin V., Wisniewski M., Droby S., Sionov E. (2019). Shifts in the composition of the microbiota of stored wheat grains in response to fumigation. Front. Microbiol..

[27] Paulson J.N., Stine O.C., Bravo H.C., Pop M. (2013). Differential abundance analysis for microbial marker-gene surveys. Nat. Methods.

[28] Layeghifard M., Hwang D.M., Guttman D.S. (2018). Constructing and Analyzing Microbiome Networks in R. Methods in Molecular Biology.

[29] McMurdie P.J., Holmes S. (2013). phyloseq: An R Package for Reproducible Interactive Analysis and Graphics of Microbiome Census Data. PLoS ONE.

[30] Wu B., Hussain M., Zhang W., Stadler M., Liu X., Xiang M. (2019). Current insights into fungal species diversity and perspective on naming the environmental DNA sequences of fungi. Mycology.

[31] Muzhinji N., Truter M., Woodhall J.W., van der Waals J.E. (2015). Anastomosis Groups and Pathogenicity of Rhizoctonia solani and Binucleate Rhizoctonia from Potato in South Africa. Plant Dis..

[32] Zida E., Neya B., O’Hanlon K., Deleuran L., Wulff E., Lund O., Shetty P., Boelt B. (2014). Fungal endophytes of sorghum in Burkina Faso: Occurrence and distribution. African J. Microbiol. Res..

[33] Rajini S.B., Nandhini M., Udayashankar A.C., Niranjana S.R., Lund O.S., Prakash H.S. (2020). Diversity, plant growth-promoting traits, and biocontrol potential of fungal endophytes of Sorghum bicolor. Plant Pathol..

[34] Tedersoo L., Bahram M., Põlme S., Kõljalg U., Yorou N.S., Wijesundera R., Ruiz L.V., Vasco-Palacios A.M., Thu P.Q., Suija A. (2014). Global diversity and geography of soil fungi. Science.

[35] Rodriguez R.J., White J.F., Arnold A.E., Redman R.S. (2009). Fungal endophytes: Diversity and functional roles. New Phytol..

[36] Agler M.T., Ruhe J., Kroll S., Morhenn C., Kim S.-T., Weigel D., Kemen E.M. (2016). Microbial Hub Taxa Link Host and Abiotic Factors to Plant Microbiome Variation. PLOS Biol..

[37] Hooper D.U., Bignell D.E., Brown V.K., Brussard L., Dangerfield J.M., Wall D.H., Wardle D.A., Coleman D.C., Giller K.E., Lavelle P. (2000). Interactions between Aboveground and Belowground Biodiversity in Terrestrial Ecosystems: Patterns, Mechanisms, and Feedbacks. Bioscience.

[38] Wardle D.A., Bardgett R.D., Klironomos J.N., Setälä H., Van Der Putten W.H., Wall D.H. (2004). Ecological linkages between aboveground and belowground biota. Science.

[39] Berendsen R.L., Pieterse C.M.J., Bakker P.A.H.M. (2012). The rhizosphere microbiome and plant health. Trends Plant Sci..

[40] Berlanas C., Berbegal M., Elena G., Laidani M., Cibriain J.F., Sagües A., Gramaje D. (2019). The fungal and bacterial rhizosphere microbiome associated with grapevine rootstock genotypes in mature and young vineyards. Front. Microbiol..

[41] Zheng Y., Gong X. (2019). Niche differentiation rather than biogeography shapes the diversity and composition of microbiome of Cycas panzhihuaensis. Microbiome.

[42] da Silva L.L., Veloso T.G.R., Manhães J.H.C., da Silva C.C., de Queiroz M.V. (2020). The plant organs and rhizosphere determine the common bean mycobiome. Brazilian J. Microbiol..

[43] Qian X., Duan T., Sun X., Zheng Y., Wang Y., Hu M., Yao H., Ji N., Lv P., Chen L. (2018). Host genotype strongly influences phyllosphere fungal communities associated with Mussaenda pubescens var. alba (Rubiaceae). Fungal Ecol..

[44] De Deyn G.B., Van Der Putten W.H. (2005). Linking aboveground and belowground diversity. Trends Ecol. Evol..

[45] Pineda A., Soler R., Pozo M.J., Rasmann S., Turlings T.C.J. (2015). Editorial: Above-belowground interactions involving plants, microbes and insects. Front. Plant Sci..

[46] Hodgson S., de Cates C., Hodgson J., Morley N.J., Sutton B.C., Gange A.C., Alan Gange C.C. (2014). Vertical transmission of fungal endophytes is widespread in forbs. Ecol. Evol..

[47] Wiewióra B., Żurek G., Pañka D. (2015). Is the vertical transmission of Neotyphodium lolii in perennial ryegrass the only possible way to the spread of endophytes?. PLoS ONE.

[48] Frank A., Guzmán S.J., Shay J. (2017). Transmission of Bacterial Endophytes. Microorganisms.

[49] Summerell B.A., Laurence M.H., Liew E.C.Y., Leslie J.F. (2010). Biogeography and phylogeography of Fusarium: A review. Fungal Divers..

[50] Leblanc N., Kinkel L., Kistler H.C. (2017). Plant diversity and plant identity influence Fusarium communities in soil. Mycologia.

[51] Leslie J.F., Zeller K.A., Lamprecht S.C., Rheeder J.P., Marasas W.F.O. (2005). Toxicity, pathogenicity, and genetic differentiation of five species of Fusarium from sorghum and millet. Phytopathology.

[52] Chala A. (2019). Genetic diversity among Fusarium species associated with sorghum stalk rot in Southern Ethiopia. Afr. J. Biotechnol..

[53] Funnell-Harris D.L., O’Neill P.M., Sattler S.E., Yerka M.K. (2016). Response of sweet sorghum lines to stalk pathogens fusarium thapsinum and Macrophomina phaseolina. Plant Dis..

[54] Robles-Yerena L., Ayala-Escobar V., Leyva-Mir S.G., Lima N.B., Camacho-Tapia M., Tovar-Pedraza J.M. (2019). First report of Cladosporium cladosporioides causing leaf spot on tomato in Mexico. J. Plant Pathol..

[55] Rai M.K., Tiwari V.V., Irinyi L., Kövics G.J. (2014). Advances in Taxonomy of Genus Phoma: Polyphyletic Nature and Role of Phenotypic Traits and Molecular Systematics. Indian J. Microbiol..

[56] Oliveira R.C., Goncalves S.S., Oliveira M.S., Dilkin P., Mallmann C.A., Freitas R.S., Bianchi P., Correa B. (2017). Natural occurrence of tenuazonic acid and Phoma sorghina in Brazilian sorghum grains at different maturity stages. Food Chem..

[57] Kalaria R.K., Patel A., Desai H. (2020). Isolation and characterization of dominant species associated as grain mold complex of sorghum under south Gujarat region of India. Indian Phytopathol..

[58] Zhao Y.-Q., Shi K., Zhang L.-J., Zhang D.-M., Yu X.-Y., Dong Y.-Y., Bi W.-B., Yu H.-R. (2016). First Report of Alternaria alternata Causing Leaf Spot on Sorghum in China. Plant Dis..

[59] Prom L.K., Cuevas H.E., Ahn E., Isakeit T., Rooney W.L., Magill C. (2020). Genome-wide association study of grain mold resistance in sorghum association panel as affected by inoculation with Alternaria alternata alone and Alternaria alternata, Fusarium thapsinum, and Curvularia lunata combined. Eur. J. Plant Pathol..

[60] Tarekegn G., McLaren N.W., Swart W.J. (2006). Effects of weather variables on grain mould of sorghum in South Africa. Plant Pathol..

[61] Kruse J., Dietrich W., Zimmermann H., Klenke F., Richter U., Richter H., Thines M. (2018). Ustilago species causing leaf-stripe smut revisited. IMA Fungus.

[62] Wollenberg T., Schirawski J. (2014). Comparative Genomics of Plant Fungal Pathogens: The Ustilago-Sporisorium Paradigm. PLoS Pathog..

[63] Prom L.K., Perumal R., Cissé N., Little C.R. (2014). Evaluation of Selected Sorghum Lines and Hybrids for Resistance to Grain Mold and Long Smut Fungi in Senegal, West Africa. Plant Health Prog..

[64] Moharam M.H.A., Stephan D., Koch E. (2018). Evaluation of plant-derived preparations and microorganisms as seed treatments for control of covered kernel smut of sorghum (Sporisorium sorghi). J. Plant Dis. Prot..

[65] Abdelfattah A., Li Destri Nicosia M.G., Cacciola S.O., Droby S., Schena L. (2015). Metabarcoding Analysis of Fungal Diversity in the Phyllosphere and Carposphere of Olive (Olea europaea). PLoS ONE.

[66] Yurkov A.M. (2018). Yeasts of the soil—obscure but precious. Yeast.

[67] Abdelfattah A., Wisniewski M., Li Destri Nicosia M.G., Cacciola S.O., Schena L. (2016). Metagenomic Analysis of Fungal Diversity on Strawberry Plants and the Effect of Management Practices on the Fungal Community Structure of Aerial Organs. PLoS ONE.

[68] Carmichael P.C., Siyoum N., Chidamba L., Korsten L. (2017). Characterization of fungal communities of developmental stages in table grape grown in the northern region of South Africa. J. Appl. Microbiol..

[69] Chowdhary A., Rhandhawa H.S., Prakash A., Meis J.F. (2012). Environmental prevalence of cryptococcus neoformans and cryptococcus gattii in India: An update. Crit. Rev. Microbiol..

[70] Rong X., Mcspadden Gardener B.B. (2013). Draft Genome Sequence of Cryptococcus flavescens Strain OH182.9_3C, a Biocontrol Agent against Fusarium Head Blight of Wheat. Genome Announc..

[71] Li J., Li H., Ji S., Chen T., Tian S., Qin G. (2019). Enhancement of biocontrol efficacy of Cryptococcus laurentii by cinnamic acid against Penicillium italicum in citrus fruit. Postharvest Biol. Technol..

[72] Domka A., Rozpądek P., Ważny R., Turnau K. (2019). *Mucor* sp.-An endophyte of Brassicaceae capable of surviving in toxic metal-rich sites. J. Basic Microbiol..

[73] Lebreton A., Corre E., Jany J.L., Brillet-Guéguen L., Pèrez-Arques C., Garre V., Monsoor M., Debuchy R., Le Meur C., Coton E. (2020). Comparative genomics applied to Mucor species with different lifestyles. BMC Genom..

[74] Borve J., Stensvand A. (2003). Use of a plastic rain shield reduces fruit decay and need for fungicides in sweet cherry. Plant Dis..

[75] Yassin M.A., El-Samawaty A.R., Bahkali A., Moslem M., Abd-Elsalam K.A., Hyde K.D. (2010). Mycotoxin-producing fungi occurring in sorghum grains from Saudi Arabia. Fungal Divers..

[76] Rozpądek P., Domka A., Ważny R., Nosek M., Jędrzejczyk R., Tokarz K., Turnau K. (2018). How does the endophytic fungus Mucor sp. improve Arabidopsis arenosa vegetation in the degraded environment of a mine dump?. Environ. Exp. Bot..

[77] Rich M.K., Nouri E., Courty P.E., Reinhardt D. (2017). Diet of Arbuscular Mycorrhizal Fungi: Bread and Butter?. Trends Plant Sci..

[78] Taylor D., Walters W.A., Lennon N.J., Bochicchio J., Krohn A., Caporaso J.G., Pennanen T. (2016). Accurate Estimation of Fungal Diversity and Abundance through Improved Lineage-Specific Primers Optimized for Illumina Amplicon Sequencing. Appl. Environ. Microbiol..

[79] Aly A.H., Debbab A., Proksch P. (2011). Fungal endophytes: Unique plant inhabitants with great promises. Appl. Microbiol. Biotechnol..

