# Supplementary material for: Baseline Data of the Fungal Phytobiome of Three Sorghum (Sorghum bicolor) Cultivars in South Africa using Targeted Environmental Sequencing
Source: J Fungi (Basel). 2021 Nov 17;7(11):978. doi: 10.3390/jof7110978 (PMC8622221; doi:10.3390/jof7110978)
Supplement: Supplementary file 1 [file jof-07-00978-s001.zip › jof-1425109-supplementary.pdf]

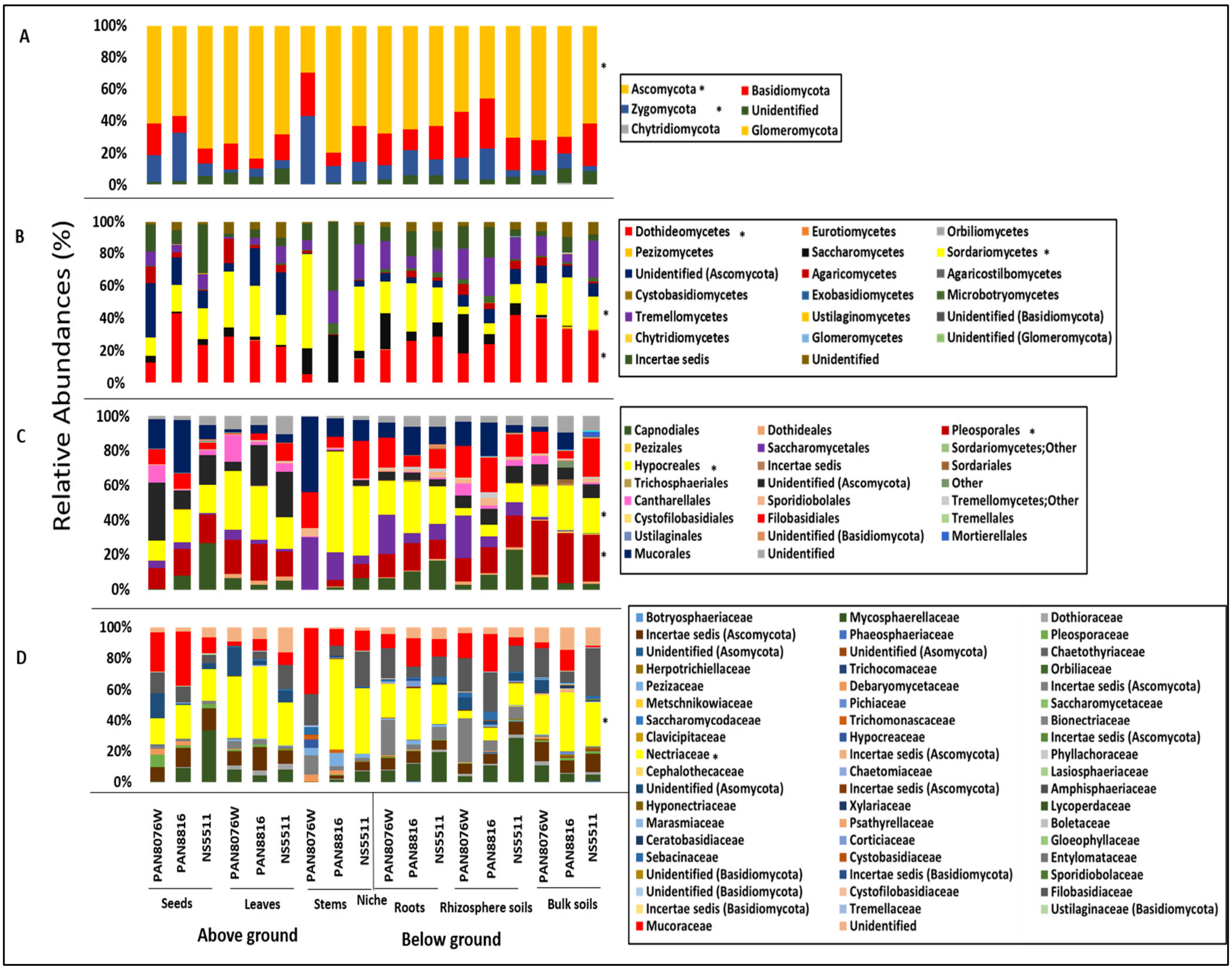

Figure S1. Bar charts of mycobiomes detected in the three sorghum cultivars (PAN8706W, PAN8816, N5511) at different taxonomic ranks: (**A**) Phyla, (**B**) Classes, (**C**) Orders, (**D**) Families. The asterisks (*) denote the mostly abundant group for each of the cultivars at the different taxonomic levels.

Table S1. Summary of the relative abundance (%) of Molecular Operational Taxonomic Units (MOTUs) taxa that were assigned at Phyla level for the different sorghum cultivars (above and below ground niche).

Phylum	Aboveground									Belowground								
	Seed			Leaves			Stems			Roots			Rhizosphere soils			Bulk soils		
	PAN8076W	PAN8816	NS5511	PAN8076W	PAN8816	NS5511	PAN8076W	PAN8816	NS5511	PAN8076W	PAN8816	NS5511	PAN8076W	PAN8816	NS5511	PAN8076W	PAN8816	NS5511
**Ascomycota***	61.6	56.9	77.5	73.9	83.4	68.2	29.8	80.1	63.2	67.6	65.3	63.2	54.4	45.7	70.3	72.1	69.8	61.5
Basidiomycota	19.7	10.7	9.5	16.5	6,7	16.5	27.0	8.2	22.6	20.3	13.2	20.6	28.7	31.9	20.4	19.0	10.4	26.7
Unidentified	1.9	2.0	5.1	7.4	5.0	10.1	0.2	1.2	2.1	3.5	5.9	6.1	3.0	3.4	4.9	6.0	9.2	8.1
Zygomycota	16.8	30.4	7.9	2.2	4.9	5.2	43.1	10.5	12.1	8.6	15.6	10.0	13.9	18.9	4.2	2.7	9.4	3.5
Chytridiomycota																0.1	0.9	0.1
Glomeromycota														01	0.1		0.2	0.1

[1] Phylum followed by an asterisk and bold indicate the most abundant in relative abundance, whilst MOTUs with zero % were left blank in the table. Names are used as assigned by the UNITE database.

Table S2. Summary of the relative abundance (%) of Molecular Operational Taxonomic Units (MOTUs) taxa that were assigned at Class level for the different sorghum cultivars (above and below ground niche).

Class	Aboveground									Belowground								
	Seed			Leaves			Stems			Roots			Rhizosphere soils			Bulk soils		
	PAN8076 W	PAN8816	NS5511	PAN8076 W	PAN8816	NS5511	PAN8076 W	PAN8816	NS5511	PAN8076 W	PAN8816	NS5511	PAN8076 W	PAN8816	NS5511	PAN8076 W	PAN8816	NS5511
**Ascomycota**																		
**Dothideomycetes** *	12.5	42.7	23.4	28.7	26.2	22.1	5.3		14.8	20.3	25.8	28.3	17.9	23.8	41.6	39.6	31.7	31.9
Eurotiomycetes							01	0.4	0.2	0.3	0.3	0.1		0.1	0.3	0.2	0.4	0.5
Orbiliomycetes									0.1							0.1	0.3	0.2
Pezizomycetes															0,1	0,1	1,1	0,3
Saccharomycetes	4.3	1.3	3.6	5.4	2.5	1.5	15.8	29.3	4.8	22.3	5.4	8.8	24.3	6.0	7.3	1.6	0.3	0.1
**Sordariomycetes** *	11.4	16.4	19.1	34.4	31.1	18.2	58.4		39.7	19.8	29.8	21.7	4.5	6.8	11.6	19.4	28.8	20.2
Unidentified	33.4	16.8	10.8	5.3	23.5	26.3	0.4		3.6	4.7	3.9	4.2	7.3	8.8	9.2	10.8	6.7	7.8
**Basidiomycota**																		
Agaricomycetes	10.5	3.0	0.4	15.3	1.8	4.6	1.5	0.5	0.5	0.8	3.9	1.9	6.9	3.7	4.9	5.3	1.0	1.1
Cystobasidiomycetes								0.1						0.1				0.1
Exobasidiomycetes																		0,1
Microbotryomycetes	02	0.2	0.7	0.3	0.7	0.9	0.5	6.3	0.5	1.9	1.4	3.4	2.7	4.4	1.1	0.9	0.8	2.5
Tremellomycetes	8.7	4.4	8.9	0.9	4.1	10.6	6.1	20.2	21.5	17.5	7.2	13.0	19.0	23.5	13.6	12.5	5.0	22.8
Ustilaginomycetes	0.3	0.9	0.6				0.1		0.1					0.1		0.1	0.2	0.1
Unidentified		0.9				0.3					0.6	2.4		0.1	0.7		0.1	01
**Chytridiomycota**																		
Chytridiomycetes																	0.5	01

**Glomeromycota**																		
Glomeromycetes															0.1		0.2	0.1
Unidentified														0.1	0.1		0.1	0.1
**Zygomycota**																		
Incertae sedis	16.8	7.9	30.4	2.2	4.9	5.2	10.5	43.1	12.1	8.6	15.6	10.0	13.9	18.9	4.2	2.7	9.4	3.5
**Unidentified**	1.9	5.1	2.0	7.4	5.0	10.1	1.2	0.2	2.1	3.5	5.9	6.1	3.0	3.4	4.9	6.0	9.2	8.1

Class followed by an asterisk and bold indicate the most abundant in relative abundance, whilst MOTUs with zero % were left blank in the table. Names are used as assigned by the UNITE database.

Table S3. Summary of the relative abundance (%) of Molecular Operational Taxonomic Units (MOTUs) taxa that were assigned at Order level for the different sorghum cultivars (above and below ground niche).

Order	Aboveground									Belowground								
	Seed			Leaves			Stems			Roots			Rhizosphere soils			Bulk soils		
	PAN8076 W	PAN 8816	NS55 11	PAN8076 W	PAN88 16	NS55 11	PAN8076 W	PAN88 16	NS55 11	PAN8076 W	PAN88 16	NS55 11	PAN8076 W	PAN88 16	NS55 11	PAN8076 W	PAN88 16	NS55 11
**Ascomycota**																		
Capnodiales	0.2	7.8	26.5	6.4	2.7	5.0		1.3	6.4	63	10.0	16.4	2.8	8.2	22.1	6.8	3.3	32
Dothideales		0.1		2.5	2.2	2.6		0.4	0.3	0.8	0.2	1.1	1.6	0.9	1.7	1.5	0.3	1.0
**Pleosporales***	12.3	15.3	16.2	19.8	21.2	14.5		3.6	8.1	13.2	15.5	10.8	13.4	14.6	17.6	30.5	27.4	26.2
Pezizales															0,1	0,1	1,0	0,3
Saccharomycetales	4.3	3.6	1.3	5.4	2.5	1.5	29.3	15.8	4.8	22.3	5.4	8.8	24.3	6.0	7.3	1.6	0.3	0.1
Sordariomycetes;Other															0,2	0,6	0,5	0,1
**Hypocreales***	11.3	19.0	16.2	34.1	30.6	17.9		57.7	39.6	19.5	28.6	21.4	4.4	6.3	10.8	16.6	24.5	19.5
Incertae_sedis				0.1	0.1					0.1	0.1					0.5	1.3	0.1
Sordariales												0.1			0.2	1.2	2.0	0.1
Trichosphaeriales		0.1			0.2	0.2		0.6			1.0				0.2		0.1	
Unidentified	33.4	10.8	16.8	5.3	23.5	26.3		0.4	3.6	4.7	3.9	4.2	7.3	8.8	9.2	10.8	6.7	7.8
**Basidiomycota**																		
Other																0,2	3,4	
Cantharellales	10.4	0.2	3.0	15.3	1.8	4.6	0.5	0.9	0.5	0.2	0.7	0.8	6.7	1.9	3.5	4.8	0.2	0.6
Sporidiobolales	0.2	0.6	0.2	0.2	0.7	0.9	5.0	0.4	0.3	1.8	1.4	3.4	2.7	4.3	1.1	0.8	0.7	2.4
Tremellomycetes;Other		0,2	0,1		0,5	0,7	0,1		0,2	0,2	1,1	1,9	0,3	3,0	0,5	0,5	0,4	1,0
Cystofilobasidiales										0,3			0,6					
Filobasidiales	8.7	8.8	4.3	0.8	3.5	9.9	20.1	6.0	21.3	17.0	5.9	11.0	18.0	20.0	12.9	11.9	4.4	21.2
Tremellales			0.1	0.1	0.1	0.1			0.1	0.0	0.1	0.1	0.1	0.5	0.1		0.2	0.6
Ustilaginales	0.3	0.6	0.9					0.1	0.1					0.1		0.1	0.1	0.1
Unidentified			0.9			0.3					0.6	2.4		0.1	0.7		0.1	0.1

**Chytridiomycota**																		
Rhizophydiales																	0.5	
Spizellomycetales																	0.1	0.1
**Glomeromycota**																		
Glomerales																	0.2	
Unidentified																	0.1	0.1
**Zygomycota**																		
Mortierellales												0,1		0,2		0,2	0,3	2,9
Mucorales	16.8	30.4	7.9	2.2	4.9	5.2	43.1	10.5	12.1	8.6	15.5	9.9	13.9	18.8	4.2	2.5	9.1	0.6
**Unidentified**																		
Unidentified	1.9	2.0	5.1	7.4	5.0	10.1	0.2	1.2	2.1	3.5	5.9	6.1	3.0	3.4	4.9	6.0	9.2	8.1

Order followed by an asterisk and bold indicate the most abundant in relative abundance, whilst MOTUs with zero % were left blank in the table. Names are used as assigned by the UNITE database.

Table S4. Summary of the relative abundance (%) of Molecular Operational Taxonomic Units (MOTUs) taxa that were assigned at Family level for the different sorghum cultivars (above and below ground niche).

**Family**	**Aboveground**									**Belowground**								
	Seed			Leaves			Stems			Roots			Rhizosphere soils			Bulk soils		
	PAN8076 W	PAN88 16	NS551 1	PAN8076 W	PAN88 16	NS551 1	PAN8076 W	PAN88 16	NS551 1	PAN8076 W	PAN88 16	NS551 1	PAN8076 W	PAN88 16	NS551 1	PAN8076 W	PAN88 16	NS551 1
**Ascomycota**																		
Mycosphaerellaceae	0.2	7.8	27.0	6.4	2.7	5.0		1.2	6.4	6.3	10.0	16.0	2.8	8.2	22.0	6.8	3.3	3.2
Dothioraceae		0.1		2.5	2.2	2.6		0.4	0.3	0.8	0.2	1.1	1.6	0.9	1.7	1.5	0.3	1.0
Pleosporales;Other	0.9	3.0	2.6	11.2	11.0	8.6		0.7	2.7	5.9	8.6	5.3	7.5	9.0	10.0	21.9	21.0	17.0
Incertae sedis	6.1	11.0	11.0	7.5	9.5	5.2		2.6	5.2	6.8	6.6	5.3	5.5	5.1	7.1	7.9	5.2	8.2
Pleosporaceae	5.3	1.2	2.3	1.1	1.1	0.6		0.4	0.1	04	0.2	0.2	0.4	0.4	0.5	0.7	1.2	0.9
Unidentified													0.1		0.2	0.5	0.7	1.3
Pezizaceae															0.1	0.1	1.0	0.3
Debaryomycetaceae	2.5	2.4	0.1	0.1	0.1	0.1	4.0	2.7	0.1	0.3	0.3	0.1	0.2	0.3		0.6		
Incertae sedis	1.4	1.1	1.1	4.3	2.2	1.2	12.7	2.3	2.0	20.6	2.8	6.6	24.0	5.5	6.3	0.8	0.2	0.1
Pichiaceae		0.1	0.1	1.0	0.1		4.8	8.4	2.6	0.8	2.3	1.4	0.1	0.2	0.7	0.1		
Saccharomycodaceae	0.3					0.1	5.4		0.1	0.5		0.7						
Trichomonascaceae							2.5	2.4	0.1	0.1								
Bionectriaceae				0.6						0.1								
**Nectriaceae***	11.3	19.0	16.0	33.3	31.0	18.0		58.0	40.0	19.4	28.0	21.0	4.4	6.3	11.0	16.5	25.0	19.5
Incertae_sedis				0.1	0.1					0.1						0.5	1.3	0.1
Sordariales;Other																0.8	0.6	
Incertae sedis		0.1			0.2	0.2		0.6		0.2	1.0				0.2		0.1	
Unidentified	33.4	10.8	17.0	5.3	23.5	26.3		0.4	3.6	4.7	3.9	4.2	7.3	8.8	9.2	10.8	6.7	7.8

**Basidiomycota**																		
Marasmiaceae					0.1			0.6		0.3	3.2	0.2	0.2	1.5	1.0			0.1
Ceratobasidiaceae	10.4	0.2	3.0	15.3	1.8	4.6	0.5	0.9	0.5	0.2	0.7	0.8	6.7	1.9	3.5	4.8	0.2	0.6
Other						0.1		0.3	0.1	0.1	0.2	0.3		0.1	0.1	0.3	0.3	0.9
Incertae sedis	0.2	0.6	0.1	0.2	0.7	0.8	5.0	0.1	0.2	1.8	1.2	3.1	2.6	4.2	1.0	0.5	0.5	1.5
Unidentified		0.1					1.3	0.1	0.2							0.1		0.1
Cystofilobasidiaceae										0.3			0.6					
Filobasidiaceae	8.7	8.8	4.3	0.8	3.5	9.9	20.1	6.0	21.3	17.0	5.9	11.0	18.0	20.0	12.9	11.9	4.4	21.2
Ustilaginaceae	0.3	0.6	0.9					0.1	0.1					0.1		0.1	0.1	0.1
Unidentified			0.9			0.3					0.6	2.4		0.1	0.7		0.1	0.1
**Chytridiomycota**																		
Rhizophydiales																	0.5	
Spizellomycetales																	0.1	0.1
**Glomeromycota**																		
Glomerales																	0.2	
Unidentified																	0.1	0.1
**Zygomycota**																		
Mortierellaceae												0.1		0.2		0.2	0.3	2.9
Mucoraceae	16.8	30.4	7.9	2.2	4.9	5.2	43.1	10.5	12.1	8.5	15.4	9.9	13.9	18.8	4.2	2.5	8.9	0.6
Unidentified	1.9	2.0	5.1	7.4	5.0	10.1	0.2	1.2	2.1	3.5	5.9	6.1	3.0	3.4	4.9	6.0	9.2	8.1

Family followed by an asterisk and bold indicate the most abundant in relative abundance, whilst MOTUs with zero % were left blank in the table. Names are used as assigned by the UNITE database.
